# Socialisation scenes in the health behaviour of teacher students at different levels of teacher training

**DOI:** 10.3389/fspor.2024.1504214

**Published:** 2025-01-06

**Authors:** Marianna Moravecz, Karolina Eszter Kovács, Bettina Kozma

**Affiliations:** ^1^ University of Nyíregyháza Institute of Physical Education and Sport Sciences, Nyíregyháza, Hungary; ^2^ Department of Counselling, Developmental and School Psychology, University of Debrecen Institute of Psychology, Debrecen, Hungary

**Keywords:** socialisation scene, health behaviour, teacher training, higher education, pedagogy students

## Abstract

The issue surrounding sport and health as valuable categories spans across generations. It is now widely recognised that inherited, lifestyle, and environmental factors influence an individual's health. Our study investigated the impact of family as the primary area of socialisation and school as the secondary area. Data collection commenced online in the spring of 2020, focusing on pedagogical students from the University of Nyíregyháza (*N* = 194). Among our research sample, kindergarten teachers exhibited the lowest indicators regarding risk behaviour. Conversely, students specialising in teaching demonstrated the most favourable outcomes regarding physical activity, with a rate of 20.6%. The family's influence presents a significant effect in both positive and negative aspects. When families prioritise instilling a love for sports, students tend to have a more favourable view of their health and aspire to embody exemplary values in the future; however, the family also presents a less encouraging image. Analysing the impact of residence reveals that a greater percentage of individuals from immigrant backgrounds identified the family as their role model (27.2%) compared to their counterparts residing in the county seat, while the influence of the teacher's personality was minimal. These findings align with earlier research. The students participating in our study hailed from the underprivileged Northern Great Plain region, which contributes to an increased search for security. The research indicates that family emerged as a significant example of values, and the objective is to foster positive health behaviours in both areas equally.

## 1 Introduction

Society's vision for the future can be influenced by the values of the young generation related to lifestyle and health awareness. Today, we are witnessing a crisis in these value preferences (1). According to the 2017 Eurobarometer survey, 9% of the population in Hungary do regular physical activity, while 53% do not do any sport at all. This inactivity rate generates several health problems, such as the development of non-communicable diseases (diabetes, obesity, cardiovascular diseases, etc.) (2). For this reason, physically active lifestyles have gained great importance nowadays (3). Socialisation settings have the most significant influence on developing this health-conscious lifestyle. The importance of the parental model and the family (as the primary socialisation arena) is unquestionable, but it is also essential to highlight the role of teachers who provide a model for students in schools (as the secondary socialisation arena). A surprising result is the low influence of physical education teachers on the health-conscious behaviour of pupils (4).

The most important task of schools, apart from the transfer of knowledge, is the kind of physical and health education that can lay the foundations for a health-conscious lifestyle among young people (5). Within the school system, institutions of higher education provide the last opportunity for physical activity in an organised setting (6). The students examined came to higher education from the disadvantaged region of the Northern Great Plain (7). In this region, it has already been shown at the secondary school level that the family background index and academic achievement show the lowest picture compared to the national level (8). An important factor is the expansion and creation of expertise of teachers and student teachers, which is essential for quality education (9).

Our research aims to map students’ health behaviour at the University of Nyíregyháza in the light of socialisation settings. The physical activity and health consciousness of students are influenced by several factors (10, 11), but due to scope limitations, we only investigated the role of family and school (teacher) as primary and secondary socialisation levels. In our study, we explored the concepts of health, health behaviour and socialisation arenas based on the theoretical background of Bakacs et al's. (12) research, which we present in our own modification. The starting point was the health behaviour rate, which is worse in Hungary than in Europe (13). Health-conscious behaviour includes prevention, health preservation and avoidance of harmful addictions (14, 15). Changing negative health behaviour patterns is a topical issue today, and coordinated work in different socialisation arenas is essential. It is essential to increase the impact of educational institutions on health behaviour, which can contribute to changing students’ healthy attitudes towards life (16). Due to its social embeddedness, healthy behaviour can be achieved by bringing about complex changes through cooperation between educational settings (family, school) and the value of the teacher as an example.

In our study, we identified three research questions, the first of which investigated health behaviours and for which we formulated two hypotheses:

• We hypothesise that, in terms of level of education, the health risk behaviours (drugs, alcohol, smoking) of students in kindergarten and primary school teacher training show a more positive picture than those of students in secondary teacher education.
• In terms of preventive health behaviour (physical activity), secondary teacher students show a more positive pattern than students in kindergarten and primary teacher education.

In the second research question block, we examined the socialisation context, also formulating two hypotheses:

• We hypothesised that students whose parents considered it important to show a positive example concerning the love of sport would have better subjective health and positive perceptions of their own health behaviour, regardless of their type of education.
• It was assumed that, regardless of the type of education, students who did not consider their own health behaviour as exemplary were not surrounded by a positive pattern of sporting activity and that the teacher (kindergarten, primary and secondary teacher) had a negative influence on their development.

The third set of questions examined the role of place of residence through a hypothesis:

• Looking at the proportion of sportspeople in the students’ immediate environment, the role of the family is more important in terms of socialisation among students living in small towns (villages, small towns, farms) than among students living in the county capital.

This study aims to address previous research gaps and comprehensively describe health-related behaviours of kindergarten, primary and secondary teacher training students from the Northern Great Plain region. In studying the particular group, the research provides insights into how early socialisation and educational settings influence health behaviours in a socioeconomically disadvantaged context. Unlike previous studies which emphasise on the positive roles of both the family and school for health behaviour development, this study offers a different perspective. It examines the dual role of family as both a positive and negative socialization force and investigates the underwhelming impact of teachers on students’ health behaviours, as reported in prior studies. By addressing these critical gaps, the study enhances the academic discourse on health socialization and offers actionable insights for improving health education in teacher training programs. The findings are intended to prepare future teachers to become people who actively promote healthy lifestyles among their pupils, thus extending the reach of their work over the long term.

## 2 Material and methods

### 2.1 Participants

The data was collected at the University of Nyíregyháza among students participating in kindergarten, primary and secondary teacher education. The survey was started in spring 2020. The number of participants is 194 (*N* = 194), of which 140 are women, and the remaining 54 are men, which is a good representation of the feminisation of the teaching profession. Looking at the distribution by training field, there are almost equal proportions of students studying in kindergarten (*N* = 64; 33%), primary (*N* = 67; 34.5%) and secondary (*N* = 63; 32.5%) teacher training. Regrading the gender distribution, 72.2% of the respondents were female and 27.8% were male. Concerning the year of education, first graders are represented in the highest ratio (34%) and fourth graders in the lowest (18%), while the ratio of second graders (26%) and third graders (22%) is almost equal. Regarding the type o settlement, the largest proportion of respondents comes from villages (37.1%) or small towns (33.5%), which may also be due to the disadvantaged eastern region. 19.1% lives in a county seat, 5.2% in a big city, 4.6% in the capital and 0.5% in a farm. Concerning the frequency of regular physical activity, the highest proportion was once or twice per week at 31%. A high ratio (40%) of physical inactivity (several times a month and below) was detected, which according to the WHO is the fourth most common risk factor for the development of disease in the world. More than half of respondents pursue individual sports (51%) and less than one-quarter play team sports, but 32% of students said they do not play any sports.

### 2.2 Instruments

In our research, we focused on the examination of physical activity, subjective health status, and health risk behaviours and examined the indicators of sporting activities brought by the family. The regulation of physical activity as measured by the question “*How often are you involved in physical activity?*” where participants could choose one option (never, once/twice per year, once/twice per month, several times per month, once/twice per week, three or more times per week). Subjective health status was measured by a 4-point Likert scale (poor, average, good, excellent) for the question “*How could you evaluate your health status?”*. Concerning the health risk behaviours, participants were asked whether the *various activities were true or false for them*, including substance try-out, denying substance use, never consuming alcohol, sometimes little alcohol consumption, sometimes heavy alcohol consumption, often little alcohol consumption, frequent heavy alcohol consumption, never smoking, occasionally smoking, smoking one pack of cigarettes per week, smoking several packs of cigarettes per week. Concerning the relevance of the role models, participants were asked whether *their love of sport* comes from their famil and/or their kindergarten/primary school/secondary school teachers. Students also had to reply whether it is true of false for them, *having any positive sporting behaviour in their environment* (including their family, best friends and teachers) and/or being negatively influenced by their kindergarten/primary school/secondary school teacher.

### 2.3 Statistical analysis

An online survey was used for data collection, and due to the COVID-19 pandemic, the questionnaire was available online only. Data obtained were analysed using SPSS 16.0 software (17), including analysis of variance, two-sample *t*-test, Chi-square test and cross-tabulation analysis.

## 3 Results

**Figure 1:**
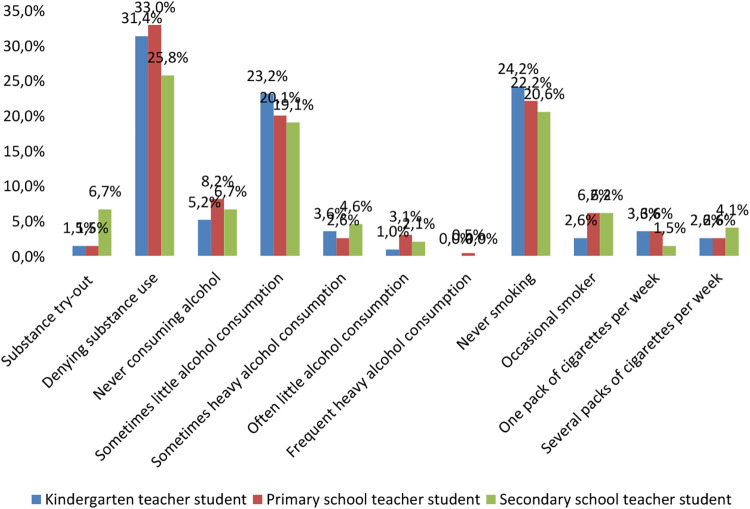
Health-risk behaviour by major (%, *N* = 194).

Concerning the risk behaviour, the health-damaging behaviour of kindergarten teacher students is generally lower. Concerning substance abuse, students in kindergarten and teacher education showed a more positive picture compared to students in secondary teacher education, while this more positive picture is not evident for alcohol and smoking (Figure 1). When looking at the health behaviour of student teachers, the most positive picture was found for kindergarten teachers. The frequency of substance try-out was higher among secondary school teachers while denying substance use was more frequent among kindergarten and primary school teacher students. Consuming more alcohol is rather typical among secondary school teacher students too, and the same could be stated concerning regular smoking. Overall, we only obtained a significant result for substance abuse try-out (*p* = 0.002), while the more positive picture of kindergarten and teacher training students was not found for alcohol (*p* = 0.922) and smoking (*p* = 0.289). Therefore our hypothesis could be only partially accepted.

**Figure 2:**
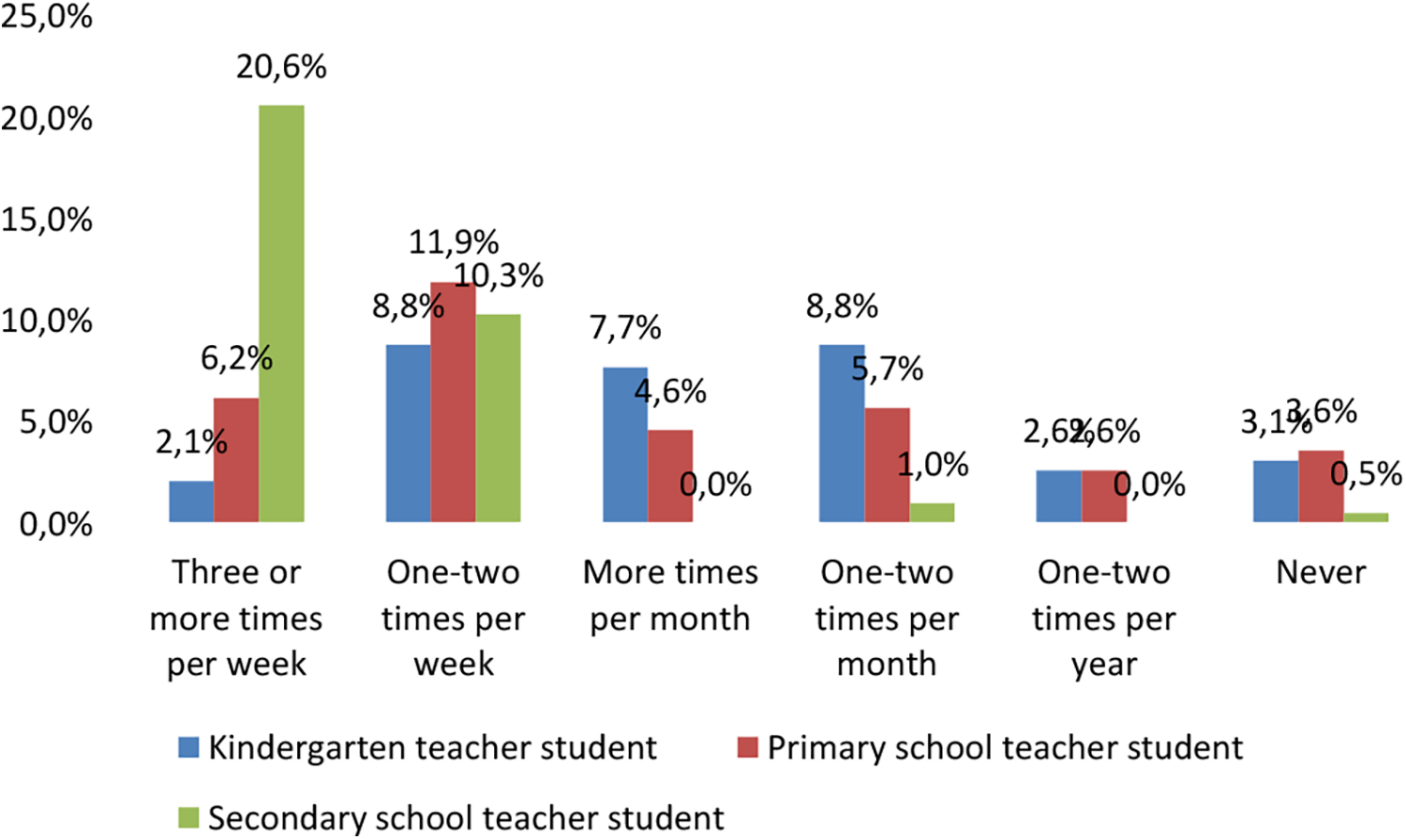
Physical activity by major (%, *N* = 194).

When examining preventive behaviour, the main focus was on comparing physical activity. The proportion of regular physical activity of at least 45 min of intensity was 20.6% for the secondary school teacher students, compared to only 8.3% for the kindergarten and primary school teacher students (Figure 2). Overall, participating in any kind of physical activities three or more times a week was the most typical among secondary school teacher students while doing sport once or twice per week was the most typical among primary school teacher students. Kindergarten teacher students reported having less physical activity.

Chi-squared test was used to test the significance value, which showed a strong significance between the variables (*p* < 0.001, Table 1).

**Table 1:** Examination of physical activity and occupations using Chi-square (*χ*^2^) test (*N* = 194).

Chi-square tests			
	Value	df	Asymp. Sig. (2-sided)
Pearson Chi-square	75,398^a^	10	0,000
Likelihood ratio	87,213	10	0,000
Linear-by-linear association	45,285	1	0,000
N of valid cases	194		

^a^ 6 cells (33,3%) have expected count less than 5. The minimum expected count is 3,25.

It is important to mention that the National Public Health Center has also prioritized and called people's attention to the importance of physical activity as a protective factor against the coronavirus (COVID-19) or any virus (18).

As I mentioned in my research, there are several aspects that determine our health behaviour, but one of the most important components is the person himself, and thus the subjective perspective he shapes (19). Therefore, it is essential to recognise our own health status, and those who follow the WHO recommendation of at least 60 min of daily physical activity (20) will also have a more positive health status.

**Figure 3:**
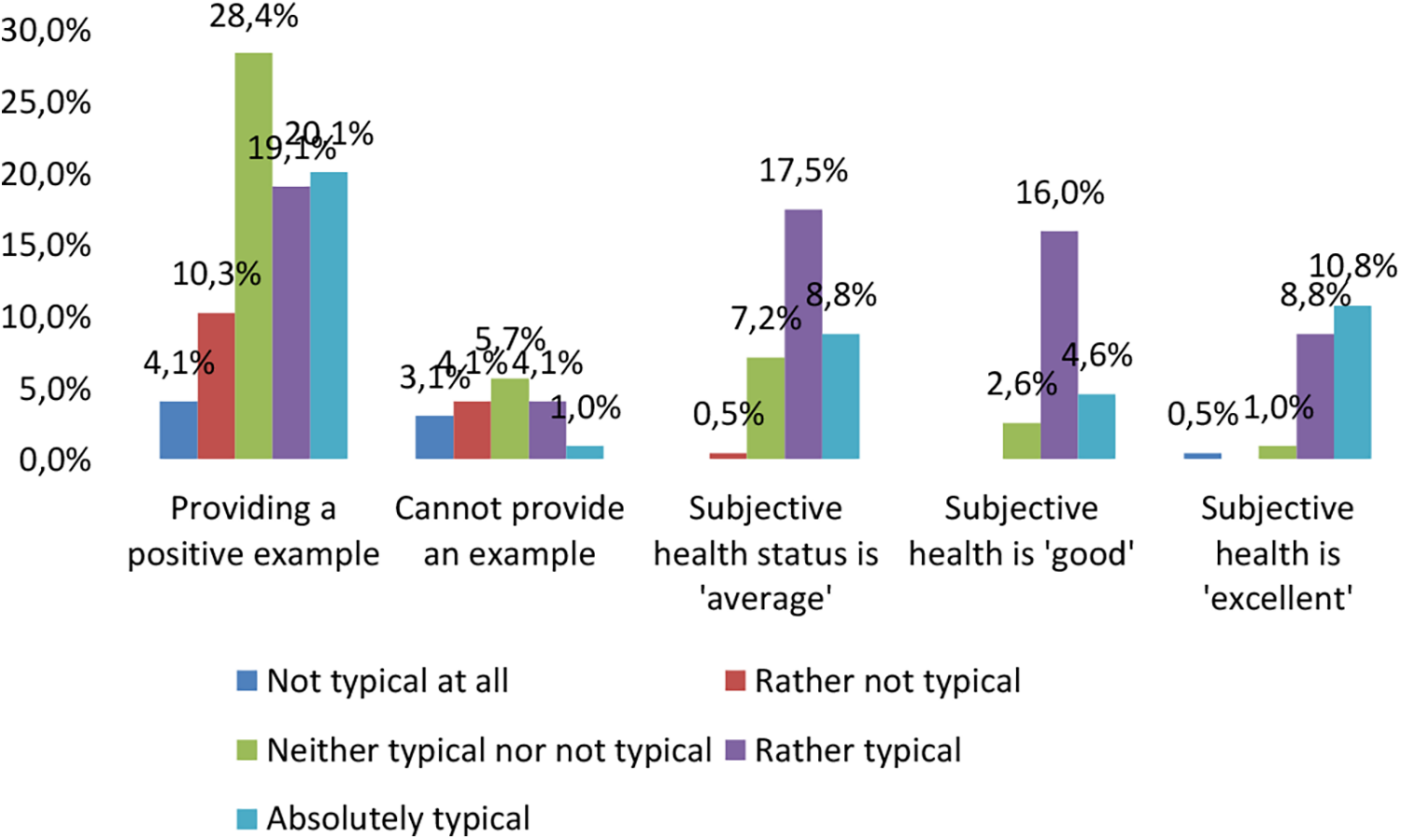
The role of taking care by the reference persons in the light of subjective health status and example value transmission (%, *N* = 194).

Regarding primary socialisation, respondents were asked to rate their love of sport coming from their family and their subjective health status on a Likert scale of 1–5 and to answer yes or no to whether they could convey their own exemplary behaviour (Figure 3). As we were interested in knowing if bringing a positive role model from the family would also make the student feel more health conscious, we considered scores of 3 and above on the scale. This means that if the family felt it was important to pass on a love of sport, then their health was rated more positively by the students. 67.6% of respondents said they would like to set an example of their own health behaviour in the future. These values were confirmed by the Anova test (Analysis of Variance), which showed a strong significance of *p* < 0.001 between the groups. This seems to confirm the positive role of the family as the primary socialisation level for health behaviour (21).

18% of students (*N* = 34) said that they would not want to set an example of healthy behaviour in the future. We examined two indicators, family environment and the teacher, and students tended to disagree with this statement. It is worth looking at the answers where the respondents answered yes; in this case, it is clear that the negative influence of the family is more strongly represented compared to the teacher. Our fourth hypothesis was not confirmed based on the results of the Dunnet test, as the students who did not want to transmit their own sport and health behaviour as an example in the future did not mention family (*p* = 0.999) and the teacher (*p* = 0.138) as the most important factors.

**Figure 4:**
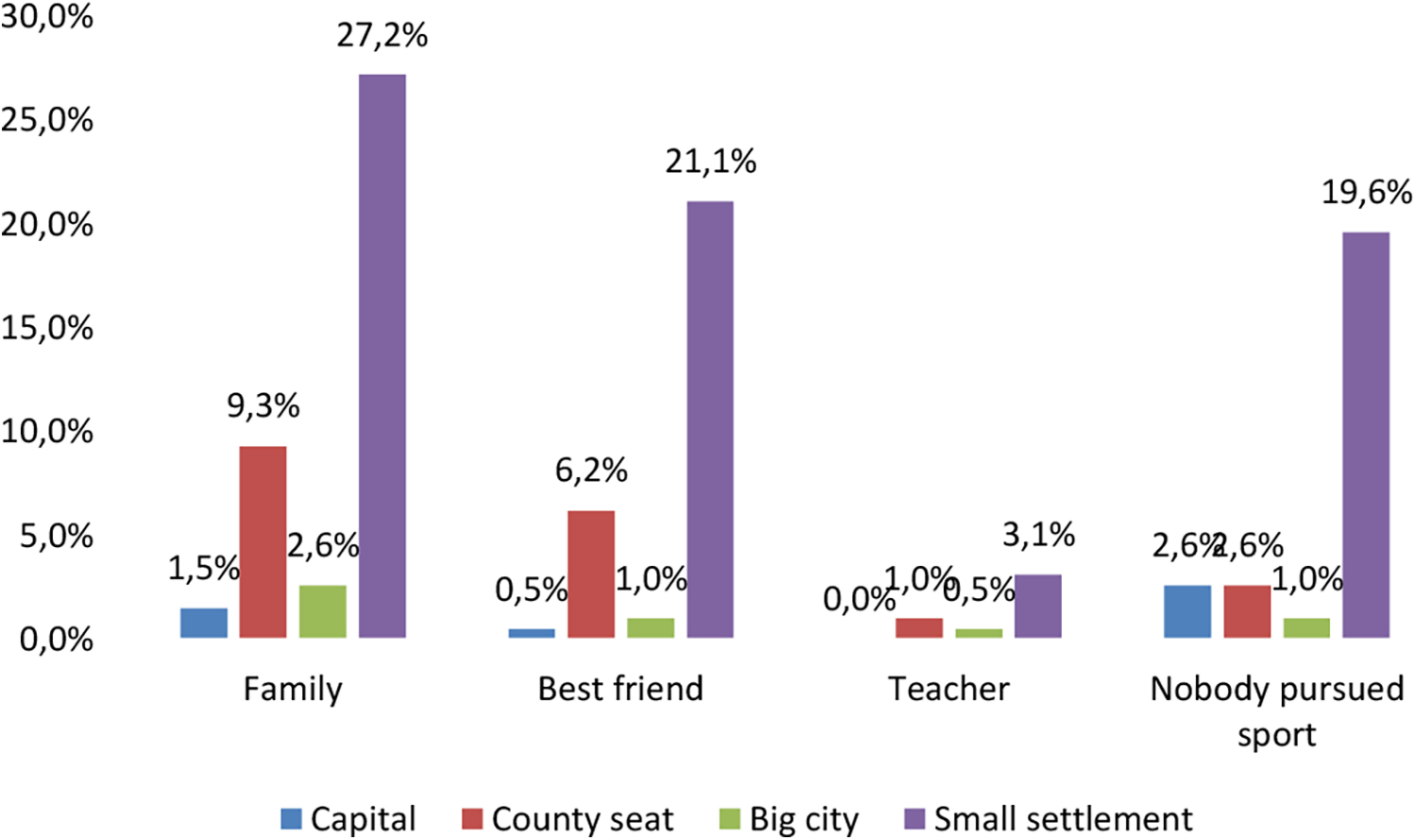
Sport examples by the type of settlement (%) *N* = 194.

It can be clearly seen that students who came from a small town indicated their family as the most frequent example of a sporting environment (27.2%). In contrast, this percentage was only 9.3% for students from a metropolitan area. Moreover, the minor role of the teacher is clearly observed, with a total of 4.6% of students stating that they had seen a teacher as a sporting role model in their immediate environment (Figure 4). The two-sample *t*-test (Table 2) showed strong significance (*p* < 0.001), i.e., students living in a small town had a higher proportion of students who mentioned family as a sporting background in their immediate surroundings compared to students living in the county seat. This finding is confirmed by a 2014 survey that found that physical education teachers pay little attention to emphasising the relationship between physical activity and health, and thus are less able to provide an example (22).

**Table 2:** Examination of the residence and sport samples using a two-sample test (*N* = 194).

		F	Sig.	t	df	Sig. (2-tailed)	Mean difference	Std. error difference	95% confidence interval of the difference	
									Lower	Upper
Did somebody pursue sport in your close environment?	Equal variances assumed	2,583,251	0,000	−8,615	192	0,000	−,57,246	,06645	−,70,354	−,44,139
	Equal variances not assumed			−13,544	127,00	0,000	−,57,246	,04227	−,65,604	−,48,888

## 4 Discussion and conclusions

Previous research has already confirmed that the physical activity and health behaviour of young people are determined by a combination of factors (10, 11). Interesting differences can be observed between the health risk behaviours of kindergarten teachers and primary and secondary school teachers, which may be due to several factors. Kindergarten teachers tend to take on a more responsible role, as they are responsible for promoting the development of young children and setting an example. As a consequence, they may be more inclined to consciously avoid behaviours that could be harmful to their health, as they know that children easily copy adult behaviour (23, 24). This may mean that kindergarten teachers are the most engaged regarding the groups surveyed. This aligns with previous studies showing that kindergarten teachers showed a higher level of commitment during the career choice process (25). On the other hand, the group of kindergarten teachers consisted exclusively of women. In contrast, the other groups studied were “spoiled” by the male participants regarding health behaviour indicators. The more positive tendency to experiment with substance use shown by kindergarten and primary school teacher students may also reflect an openness and willingness to experiment among young people (26). However, the markedly positive rates for alcohol and tobacco use are not evident, suggesting that these substances require considerable discretionary choice, influenced by deeper social norms and health risks.

In terms of preventive health behaviour (physical activity), the secondary school teacher students show a more positive pattern than the kindergarten and primary school teacher students. The chi-square test yielded a significant result, so we have retained this hypothesis. The results may be interpreted by more facts. Firstly, secondary school teacher students may have a greater enthusiasm and competitive spirit for sports, as they are often expected to lead active, exemplary lives when teaching (27). In addition, physical activity and physical education play an important role in teacher training, which can contribute to the development of sporting habits among students (28). In the case of kindergarten and primary school teacher students, the nature of the teaching work and the time constraints experienced by young people can also have an impact on the support for sporting activities. Kindergarten and primary school teacher students often have multiple responsibilities, which can reduce the time available for sport (29, 30). Overall, it is the difference between professional requirements, student engagement and leisure time schedules that affect their physical activity and preventive behaviour.

Our third hypothesis was confirmed: if the family considered it important to pass on the love of sport, the students would rate their subjective health status as better and have a positive perception of their own exemplary health behaviour. Young people are aware that they can set an example for future generations through their own behaviour and lifestyle (31). This trend is a sign of a sense of responsibility and conscious career building among young people, who want not only to preserve their own health but also to have a positive impact on the health of others. In conclusion, family background, socialisation and conscious example setting are key factors in shaping young people's health-conscious behaviour, which can contribute to improving community health in the long term (32–34). The family is the children's first socialisation environment and is considered the primary socialisation environment. Therefore, it is essential to develop the emotional and social competence of family members (parents, siblings, grandparents, and relatives), which strongly impact and influence the development of young people's personalities (35). They are the first people children see as models, from whom they learn health habits, hygiene, and commitment to physical and mental activity, and they shape self-confidence, qualities that play a prominent role in developing a good quality of life (36, 37). A sporty family provides a favourable environment for developing a child's positive attitude towards physical activity (38). The primary socialisation level is emphasised in shaping health as a value, whereby they act as role models to transmit positive values (39, 40). Based on Nagy's formulation (21), we consider the family the primary socialisation arena, and the school the secondary. The family is responsible for communication, identity and other essential behaviours. In contrast, at school, the culture of values and habits is no longer directly influenced by the family but is seen in a different light, with the help of other people (teachers, tutors, friends) (41).

Our fourth hypothesis was not confirmed. The amount of students who say they do not want to set an example through their health behaviour is a remarkable phenomenon, with several possible reasons behind it. The first is the influence of the family environment, which is a fundamental factor in shaping individuals’ values and attitudes towards health. The atmosphere at home, family habits and the role of parents as role models play a key role in shaping young people's health behaviour (42). The negative influence of the family is felt more than that of teachers may suggest that family norms and values are more closely linked to everyday life choices. Educational institutions, while also having a significant impact on students, cannot always counterbalance the weight of family influences (43). Furthermore, the relationship between teachers and the subjects taught also influences this dilemma, as if students do not find the teacher credible, this may further reduce the commitment to a healthy lifestyle represented by the teacher (44). The personal experiences of individuals, social norms and the power of the environment all contribute to how students will relate to health values in the future.

Our last hypothesis was confirmed by examining the role of place of residence. Students living in a small town had a higher proportion of students who mentioned family as a sporting background in their immediate surroundings compared to students living in the county seat. In small towns and cities, immediate family ties are often stronger and closer, so the role of parents as role models for athletes is more prominent (45, 46). For young people, the home environment provides the most important role models, so family sporting traditions can have a significant influence on students’ sporting and health-conscious behaviour. In addition, students living in county towns are often exposed to a wider range of opportunities and different cultural backgrounds. In urban environments, competition between schools and sports facilities makes sporting habits more diversified and not always linked to family traditions (47, 48). The larger population can lead to a more fragmented attitude among students, making it more difficult for direct family influences to prevail.

Overall, reflecting on the results of the research, since health behaviour is socially embedded and slow and difficult to change (49), greater effectiveness can be expected from health promotion interventions focusing on developing children by establishing their health literacy early. In this context, it is particularly important to study this area in professions whose representatives influence the health literacy of the growing generations through their behaviour and patterns of behaviour (25). International studies have found that health literacy alone does not lead to observable behavioural changes in health behaviour (50). In addition, results from Hungarostudy surveys show that longer time spent in the school system has a positive impact on health behaviour through mediating variables (49). In light of our results, it can be stated that for the population under study, subject knowledge about health and its maintenance does not guarantee applying subject knowledge to health behaviour indicators. On the contrary, the patterns and values brought from the family are decisive for health awareness.

This study has several limitations that may affect the interpretation of the findings. First, we should note the role of the geographical context. The research is carried out in a specific location (Northern Great Plain, Hungary) which rather represents disadvantaged socioeconomic status. Therefore, the findings may not be relevant to other locations with distinct cultural or economic settings. The focus of the research should also be noted since the paper primarily explores the impact of family and school on pedagogical students’ health practices. Although this emphasis is important, it may ignore other significant aspects such as peer influence, neighbourhood resources, and larger cultural norms, which all have a substantial impact on health habits. We also should note that we did not investigate how various family structures or dynamics may impact health habits differently. The cross-sectional nature of the study also can be mentioned as a limitation. Further research is necessary to offer longitudinal perspective on how health habits change over time in response to educational instruction and socialisation effects. The small sample size of the study may also restrict the findings’ generalisability. A bigger and more diversified sample may yield more reliable data and insights. The research was based on an online data collection, carried out during the COVID-19 pandemic, potentially introducing biases related to accessibility and participation. The results may be skewed as students who were less affected or engaged by the epidemic might not have taken part. The use of self-reported measurements for physical activity and health habits might result in inaccuracy owing to social desirability bias or misinterpretation of survey questions. Besides, since teachers have a modest effect on pupils’ health behaviors, further research should be conducted with a more thorough examination of the teachers’ role. This result may benefit from more investigation, particularly into how teacher training programs might be enhanced to promote better health outcomes for children. Overall, further study might improve knowledge by including a wider range of impacts and using more rigorous methodological techniques.

The research values indicated an increased search for security among the students we studied, probably due to their social background in the disadvantaged region of the Northern Great Plain. For our research sample, the results are in line with Bauer's (51) finding that the family, as a value indicator in the value-loss perception of the generation growing up after the regime change, plays a decisive role in shaping the positive (or negative) health behaviour of young people. In our study, we obtained similar results to those of Horváth's (4) research, where he found that students’ health behaviour depends, to a small extent, on the teacher's pattern. Institutions and individuals shape the value systems around physical and health education in schools, sometimes cooperating but sometimes contradicting each other, causing value conflicts in the lives of young people. Based on our research, we can conclude that in order to resolve this conflict, the aim is to increase the health behaviour of students in teacher training, as they will be the future generation who will learn in school the positive health behaviour patterns that will shape their personality in the future. In the battle of the socialisation arenas referred to in our title, the family, as an example, emerged victorious, based on the research findings. However, it turns out that the family can not only push young people's health behaviour in a positive direction but also shape negative images to a greater extent. In order to reduce this rate, it would be advisable to coordinate the socialisation arenas mentioned at the beginning of our study. It would be essential that the positive influence of both the family and the school on health behaviour be equally fulfilled.

## Data Availability

Data are available only on request due to ethical restrictions. For further information, please contact the following email address: kovacs.karolina@arts.unideb.hu.
